# District health management and stillbirth recording and reporting: a qualitative study in the Ashanti Region of Ghana

**DOI:** 10.1186/s12884-024-06272-x

**Published:** 2024-01-29

**Authors:** Nana A. Mensah Abrampah, Yemisrach B. Okwaraji, Kenneth Fosu Oteng, Ernest Konadu Asiedu, Rita Larsen-Reindorf, Hannah Blencowe, Debra Jackson

**Affiliations:** 1 Faculty of Epidemiology and Population Health, Department of Infectious Disease Epidemiology, London School of Hygiene and Tropical Medicine, London, UK; 2 Maternal, Adolescent, Reproductive & Child Health Centre, London School of Hygiene & Tropical Medicine, London, UK; 3 Ashanti Regional Health Directorate, Ghana Health Service, Kumasi, Ghana; 4 National Centre for Coordination for Early Warning and Response Mechanisms, Accra, Ghana; 5 School of Public Health, University of the Western Cape, Cape Town, South Africa

**Keywords:** Health systems, District health management, District health management teams, Stillbirths, Fetal deaths, Data systems, Quality of care

## Abstract

**Background:**

Despite global efforts to reduce maternal and neonatal mortality, stillbirths remain a significant public health challenge in many low- and middle-income countries. District health systems, largely seen as the backbone of health systems, are pivotal in addressing the data gaps reported for stillbirths. Available, accurate and complete data is essential for District Health Management Teams (DHMTs) to understand the burden of stillbirths, evaluate interventions and tailor health facility support to address the complex challenges that contribute to stillbirths. This study aims to understand stillbirth recording and reporting in the Ashanti Region of Ghana from the perspective of DHMTs.

**Methods:**

The study was conducted in the Ashanti Region of Ghana. 15 members of the regional and district health directorates (RHD/DHD) participated in semi-structured interviews. Sampling was purposive, focusing on RHD/DHD members who interact with maternity services or stillbirth data. Thematic analyses were informed by an a priori framework, including theme 1) experiences, perceptions and attitudes; theme 2) stillbirth data use; and theme 3) leadership and support mechanisms, for stillbirth recording and reporting.

**Results:**

Under theme 1, stillbirth definitions varied among respondents, with 20 and 28 weeks commonly used. Fresh and macerated skin appearance was used to classify timing with limited knowledge of antepartum and intrapartum stillbirths. For theme 2, data quality checks, audits, and the district health information management system (DHIMS-2) data entry and review are functions played by the DHD. Midwives were blamed for data quality issues on omissions and misclassifications. Manual entry of data, data transfer from the facility to the DHD, limited knowledge of stillbirth terminology and periodic closure of the DHIMS-2 were seen to proliferate gaps in stillbirth recording and reporting. Under theme 3, perinatal audits were acknowledged as an enabler for stillbirth recording and reporting by the DHD, though audits are mandated for only late-gestational stillbirths (> 28 weeks). Engagement of other sectors, e.g., civil/vital registration and private health facilities, was seen as key in understanding the true population-level burden of stillbirths.

**Conclusion:**

Effective district health management ensures that every stillbirth is accurately recorded, reported, and acted upon to drive improvements. A large need exists for capacity building on stillbirth definitions and data use. Recommendations are made, for example, terminology standardization and private sector engagement, aimed at reducing stillbirth rates in high-mortality settings such as Ghana.

**Supplementary Information:**

The online version contains supplementary material available at 10.1186/s12884-024-06272-x.

## Background

Every year, 1.9 million babies are stillborn [1]. Despite this number, stillbirths are rarely discussed in global and national conversations on improving reproductive, maternal and newborn outcomes [2, 3]. The World Health Organization (WHO) *International Classification of Diseases (ICD), Edition 11* defines stillbirth as a baby born with no signs of life at 22 or more completed weeks of gestation [4]. Stillbirths are categorized by timing of fetal death in relation to the onset of labour [4]. Most intrapartum fetal deaths, occurring during labor, and many antepartum fetal death, before the onset of labor, can be prevented with strong health systems [2]. While strengthening health systems broadly is key, increased attention needs to be placed on the health workforce [5–9]. Involving health workers in stillbirth recording and reporting is fundamental for collecting accurate data, understanding causes and risk factors and driving effective public health interventions.

The district health system has long been seen as the foundation of strong health systems [10]. In the realm of public health, District Health Management Teams (DHMTs) play a pivotal role as drivers of health initiatives. DHMTs are responsible for effectively planning and budgeting, human resource management, monitoring service quality, and resource allocation to support health facilities and meet needs of the population within their comunities [11]. The dedication of DHMTs to ensuring the well-being of their communities extends to the comprehensive recording and reporting of health indicators, including the critical aspect of stillbirths [12].

DHMTs serve as the cornerstone of data collection, analysis, and reporting mechanisms within their respective districts [12]. In many low- and middle-income countries (LMICs), including Ghana, information on stillbirth is collected at the facility-level and entered into routine health information systems at the district-level. Common bottlenecks reported for using maternal and newborn health data have included weak staff capacity for data management and use (interpretation, analysis, and planning) [13]. Specifically related to stillbirths, existing literature highlights the challenges that impede the quality and availability of stillbirth data. These factors relate to omission and classification of stillbirth, low levels of understanding and engagement on stillbirth issues, and inconsistent application of stillbirth definitions [14].

With the release of the global stillbirth report by the United Nations Children’s Fund (UNICEF) and WHO, evidence suggests that measures to improve accuracy of stillbirth data are needed now more than ever [2]. The UNICEF/WHO report urges countries to report and review stillbirth data locally at the facility and district, and reduce incentives for misreporting outcomes, and to monitor potential misclassification. This paper, the first in-depth analysis of district health management and stillbirths, is the first of two-papers looking at the role of DHMTs and facility-level health workers in stillbirth recording and reporting. The overall aim of this study is to understand stillbirth recording and reporting in the Ashanti Region of Ghana from the perspective of DHMTs.

## Methods

### Aim

Based on literature reviews conducted, specific objectives of the paper include: to explore the experiences, perceptions, and attitudes of DHMTs on stillbirth recording and reporting; to understand stillbirth data flow and how stillbirth data is used by DHMTs; and to explore leadership and support mechanisms available from the district-level to facilitate stillbirth recording and reporting at the facility-level.

### Region selection and study participant characteristics

The 2020 Ghana Health Service (GHS) Family Health Division Annual Report reported the stillbirth rate (SBR) for Ghana at 12.3 per 1 000 total births [15]. The SBR declined in most regions except for four regions including the Ashanti Region. The Ashanti Region reported a SBR of 12.2 per 1 000 total births, and the highest total stillbirth number across all regions (1580 stillbirths recorded for year 2020) [15]. Additionally, stillbirth related indicators, including maternal and neonatal deaths remain high for the region [15, 16].

**Fig. 1:**
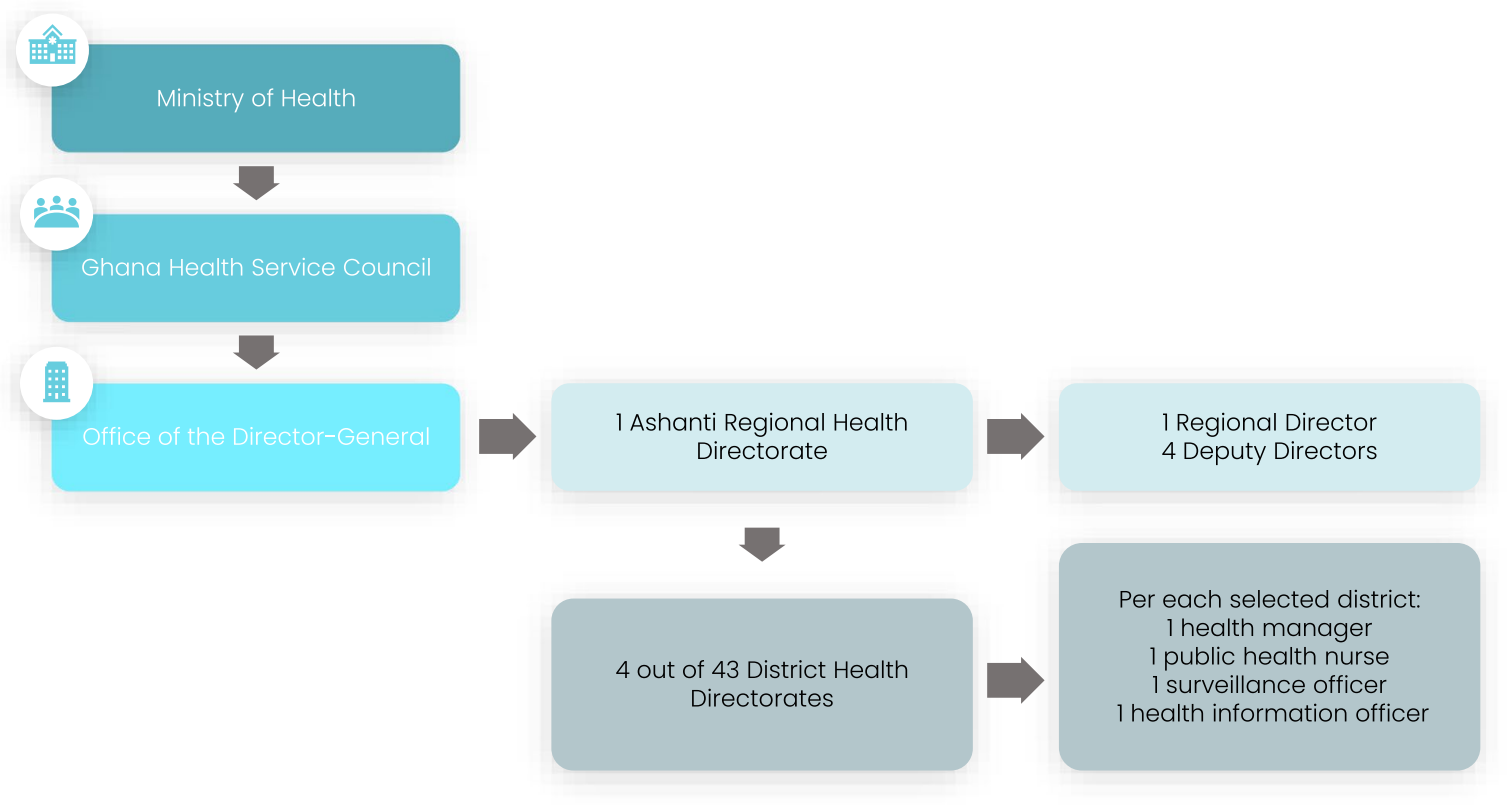
RHD/DHD organization and participants of interest to the study

Within the Ashanti Region, we selected study participants from the public/government sector. Namely, the Regional Health Directorate (RHD) and four out of the 43 District Health Directorates (DHD) were selected to represent different levels of stillbirth reporting (Fig. 1). The RHD champions the implementation and monitoring of health policies formulated by the Ministry of Health. DHDs provide leadership, supervision, management, and technical support to their sub-districts and facilities.

**Fig. 2:**
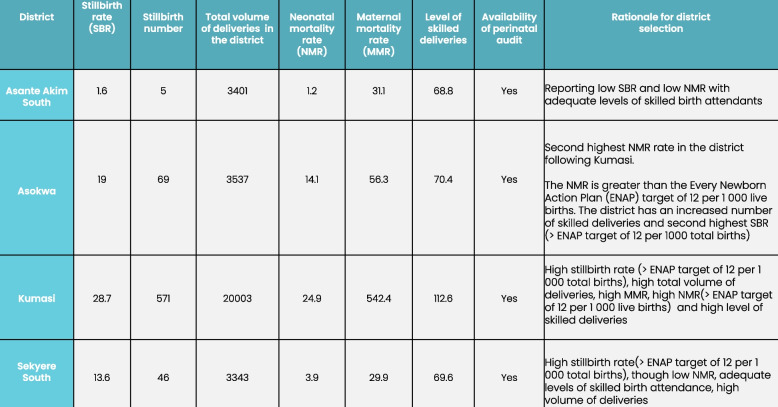
Selection criteria for study districts in the Ashanti Region, as reported in the 2021 DHIMS-2 system from the Ashanti Region

Selection of participants from the study region and districts (Fig. 2) was purposive aiming to include viewpoints from a variety of RHD/DHD cadres. All five members of the leadership team at the regional level were invited to participate. At the district level, we focused on members who interacted with maternity services or stillbirth data; aiming for each district to interview one district health manager, one public health nurse, one surveillance officer and a health information officer. In total, 21 participants were of interest to the study.

### Procedure

Semi-structured interviews were conducted to explore the experiences, perceptions, and attitudes on stillbirth recording and reporting; stillbirth data; and leadership and support mechanisms, using an interview guide (Supplementary document 1). A visual aid was developed to further elicit perspectives on stillbirth types (Supplementary document 2). The interview guide was jointly developed by the authorship team using insights gathered from the WHO reproductive, maternal, neonatal, child, and adolescent health policy survey stillbirth review [17], an analysis of existing literature, and conversations with stillbirth measurement experts. The guide was tested with the in-country team in Ghana to ensure that the questions were understood in this context. Interviews were conducted over Zoom in English by the first author, due to in-country protocols for COVID-19, time and cost-effectiveness. All interviews were recorded on Zoom, transferred into the London School of Hygiene and Tropical Medicine (LSHTM) password secured drive of the first author and transcribed verbatim. Consent forms (Supplementary document 3) were shared with participants ahead of interviews and verbal consent was obtained during interviews. Interviews were between 45 min to an hour long.

### Data collection and analysis

Thematic analyses were used for the study. This was guided by the Braun and Clark 6-step approach: familiarization with the data, generating initial codes, searching for themes, reviewing themes, defining and naming themes and report production [18].

All interviews were coded by the first author, and a random 30% reviewed by a second and third coder. When there was discrepancy between the coders, a discussion was held to address and agree on a way forward. Inductive and deductive approaches were applied. The literature review informed the selection of three major a priori themes (experiences, perceptions, attitudes on stillbirth recording and reporting; stillbirth data; and leadership and support mechanisms). Sub-themes were identified and derived from the interviews. The authorship team had access to the blinded interview transcripts to facilitate agreement on identified themes. NVivo software was used to manage and code the data.

Perspectives shared by study participants were taken at face value to highlight the realist approach to the research. The study followed the consolidated criteria for reporting qualitative studies (COREQ) guidelines [19].

## Results

Fifteen interviews were conducted (out of a total of 21), including: 3 senior managers from the regional health directorate, 4 district health managers, 3 district public health nurses, 3 district health information officers and 2 district surveillance officers. Two senior regional managers declined to participate in the interviews as they did not work directly on stillbirths. We received no responses to participate in the interview from 2 surveillance officers and 1 health information officer. One district, Asante Akim South, did not have a Public Health Nurse at the time of the interviews. The 15 study participants interviewed represented 8 women and 7 men, with an average of 10 working years.

**Fig. 3:**
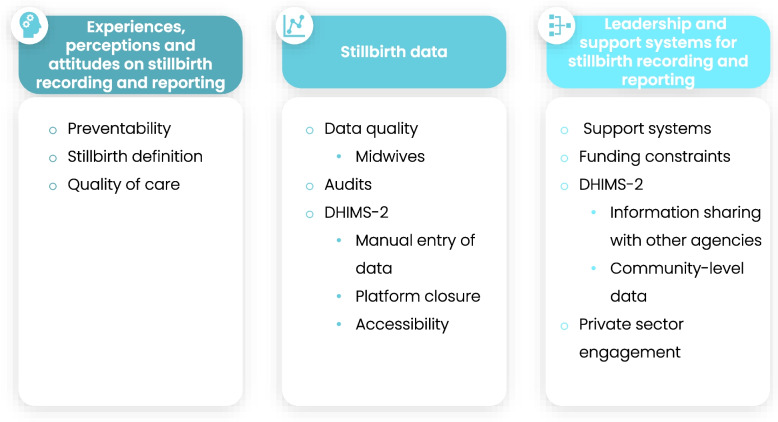
Major and sub-themes from interviews

Data saturation was achieved after 15 interviews with the following themes emerging. Ten sub-themes were identified from the interviews across the three a priori major themes. For experiences, perceptions and attitudes, sub-themes relating to preventability, stillbirth definition, and quality of care were identified. For stillbirth data, recurring themes included data quality, audits, and the district health information management system (DHIMS-2). Themes relating to leadership and support mechanisms touched on available support mechanisms, funding constraints, DHIMS-2, and private sector engagement. Figure 3 provides an overview of the major and sub-themes from the study.

### Experiences, perceptions, and attitudes on stillbirth reporting and recording

#### Preventability

All respondents were aware of the importance of preventing a stillbirth. Notably, antenatal care (ANC) and health worker skills were flagged as being critical to end preventable stillbirths.

“We educate women to come for ANC and to report any danger signs during pregnancy...there are times also when we organize in-service or refresher trainings for the midwives… (Public Health Nurse #12).”

The importance of recording stillbirths to inform interventions and course-correct actions was shared:

“The recording helps us know how we are progressing or retrogressing so that the necessary steps can be taken to correct the errors (Public Health Nurse #6).”

#### Stillbirth definition

Most respondents were aware of what a stillbirth entails, describing it as the death of a baby before or during delivery. Respondents highlighted that health facilities within the region align with the Ghana Health Service institutional definition of stillbirths.

“That one, we are working within the national Ghana Health Service. So, the definition for Ghana Health Service is what we use; we don’t have different definition (Health Information Officer #7).”

When probed further on the gestational age threshold in weeks for defining a stillbirth in Ghana, there was variation with 20 weeks (about 4 and a half months) and 28 weeks (about 6 and a half months) were commonly referenced.

“Stillbirth is a death or end of pregnancy after the 20^th^ week. After the 20^th^ week, if the pregnancy is terminated, it is stillbirth but if it is less than 20 weeks, it becomes a miscarriage (District Health Manager #8).”

“So, for us as a country, if you have a baby that is not born alive after 28 weeks of gestation, we consider it as stillbirth (Deputy Director #4).”

Other respondents characterized stillbirth as fresh or macerated, with little reference made to the gestational age of the fetus.

“The fresh is immediately the death occurs even before delivery but within the delivery process. For macerated, the child may die for let say some few days before the mother reports to the facility (Public Health Nurse #12).”

#### Quality of care

A few respondents with background in clinical care (i.e., public health nurses and members of the regional health directorate) described stillbirth in terms of antepartum and intrapartum stillbirth. Respondents equated antepartum stillbirth to before labor reflecting the quality of antenatal care and intrapartum stillbirth to during delivery reflecting the quality of delivery care.

“The antepartum (*stillbirth)*, I will say will reflect the quality of antenatal care whilst the intrapartum (*stillbirth)* reflects the quality of the delivery care (Deputy Director #2).”

Some respondents linked the outcome of stillbirth to health systems failure and health workforce skills.

“We have two types of stillbirths; we have fresh and macerated stillbirth. The fresh one has to do with technicality of the midwife in assisting the woman to deliver …. When it is macerated, we consider that it was a system problem (District Health Manager #11).”

### Stillbirth data

**Fig. 4:**
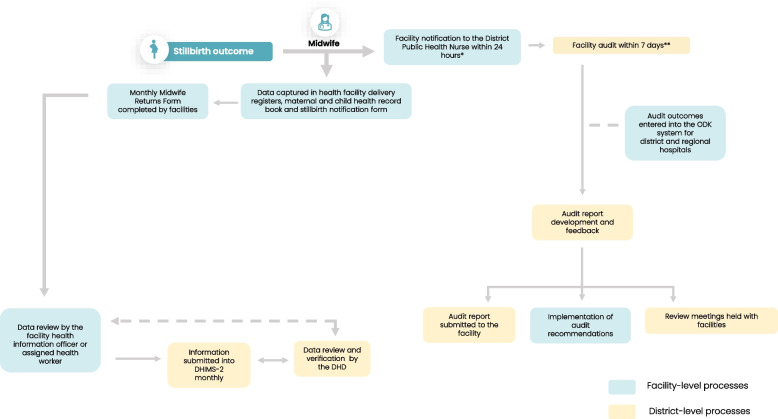
Stillbirth data flow as reported by respondents *Except for the regional hospital where an email is sent per Electronic Perinatal Death Surveillance and Response (ePDSR) system ** Performed by a multi-disciplinary team, including health workers at the facility and the DHD

Figure 4 illustrates the flow of stillbirth data from the facility-level to the district-level as described by the respondents.

When a stillbirth occurs, notification is sent to the district public health nurse. Data capture for stillbirth is manually entered into a maternity registry. At the end of the month, stillbirth data at the facility is tallied and entered into the paper-based Midwife Returns Form (also known as Form A), a form which captures key data on pregnancy outcomes. The information on the Midwife Returns Form is then transferred into the electronic data management system, DHIMS-2, the database for storing health service data in Ghana. For the district and regional hospitals, stillbirth data is also captured into the Open Data Kit (ODK) system. The ODK system is the national maternal/perinatal audit form translated into an online form. The ODK system requires health workers to provide further information on the circumstances surrounding a stillbirth.

Most respondents shared that data quality checks, audits, and the DHIMS-2 data entry and review, are primary functions played by the district health management teams.

#### Data quality

Data quality checks occur both at the facility and district-level. Respondents shared that data validation meetings with health facilities help to ensure that data being processed is accurate.

“We invite the facility heads for data validation meetings at the DHD every month. During the validation meetings, we do analysis of the data (Health Information Officer #7).”

When inconsistencies are reported in the data, the DHD schedules a meeting with the facility to review the paper-source documents and make corrections where needed.

“It should not be less or more. Once it is less or more, there is a data quality issue, which means that one is omitted or an additional one is smuggled. We need to go back to the facility and look at the data and make the necessary correction (Surveillance Officer #5).”

Inaccurate reporting linked to internet connectivity for DHIMS-2 was also reported.

“Sometimes the DHIMS-2 can also go off temporarily. If you do not follow up to see that what you have entered, it will not be recorded (District Health Manager #10).”

#### Midwives

The majority of respondents flagged that midwives were responsible for many data quality gaps in stillbirth data. These were reported to be both deliberate omissions, for example to avoid blame:

“The midwife will decide not to capture a stillbirth because sometimes, maybe they are running from their responsibility... maybe the death was due to the inaction of the health worker. That is what I can say (Health Information Officer #4).”

Or from errors in classification:

“We realize that certain deaths are captured as stillbirths, meanwhile the baby was out for some period before the baby passed out and that is certainly not a stillbirth. So that misjudgment on the part of the midwife recording is there (District Health Manger #9).”

Lack of recording may also occur due to increased workload:

“…For example, the midwife may be busy and forget to call when a stillbirth happens, and it is only during data verification that we discover (Surveillance Officer #14).”

#### Audits

The importance of audits was highlighted strongly by all respondents. Some respondents shared that audits take place if the fetus is over 28 weeks.

“Of course, if you are born at 27 weeks and you make it, we take you but if you are below 28 weeks and you do not make it, nobody will audit that death. You are not required by the service (GHS) to audit that death because the health system is not robust to be able to take care of such babies (Deputy Director #4).”

#### District health information management system-2

The importance of the online DHIMS-2 for review and analysis of stillbirth data was universally shared. Respondents mostly discussed gaps when the DHIMS-2 was discussed.

#### Manual entry of data

Stillbirth data is originally captured on paper before being transferred into the DHIMS-2, which was reported to lead to potential data entry errors. With manual entry, respondents reported that the midwife attending to the labor records the birth outcome on paper before it is transmitted by the facility/district health information officer to the DHIMS-2.

“The midwife will do the recording on the paper, then send to HI (Health Information) Officer. As to whether the HI is entering the real data into DHIMS-2, we are not sure. I think that one is a challenge for us (Public Health Nurse #12).”

A lack of understanding the terminology associated with stillbirths was also flagged:

“During review meetings, the midwives will say no this is a wrong figure. Sometimes, the health information officer may not understand some of our midwifery terms. The health information officer may enter it wrongly (District Health Manager #8).”

#### Platform closure

At the end of each month, DHIMS-2 is closed 60 days after that month ends. This is done for data verification at the district level. Health facilities do not have access to record or review the data in DHIMS-2 after 60 days when locked. If a facility failed to input the data before DHIMS-2 is locked, some respondents reported they experienced health facilities adding the data to the next month.

“If a particular month, a facility is to report, and DHIMS-2 is locked… In DHIMS-2, you realize this facility did not have any stillbirth for that month. You call the facility, and you realize that yes, they (health workers) recorded a stillbirth in their register, but it wasn’t entered in the DHIMS-2 because DHIMS-2 was closed. The following month when DHIMS-2 is open, they(health workers) add it to the new month (District Health Manager #10).”

#### Accessibility

Accessing the information in the DHIMS-2 is only available to health information officers, and senior managers within the public health system. Respondents shared this limits engagement of health workers in stillbirth data use.

“It is only the health information officer in the district who has access to the DHIMS-2. I think it is very challenging. Ideally even the midwife who is using the data should be able to access and engage with DHIMS-2 … (Public Health Nurse #12).”

### Leadership and support mechanisms

#### Support systems

All respondents shared that the RHD/DHD are committed to improving stillbirth recording and reporting. Specifically, over half of interviewed respondents mentioned the role of audits as a sign of leadership commitment to reducing stillbirths.

“Yes, we are committed because whenever you record any stillbirth you have to find out from the midwives, is it really a stillbirth? That is why we have the audit.…we are committed to reducing stillbirths (Health Information Officer #7).”

Additionally, feedback and introduction of the DHIMS-2 and the ODK system were seen by respondents as a sign of leadership commitment to stillbirth recording and reporting.

Specifically, the ODK system is a regional initiative introduced by the RHD to obtain timely information on stillbirth and the mother following an audit.

“The ODK …is a regional initiative. It captures everything that was supposed to be captured for the perinatal audit, just that it is electronic. By the time a facility has finished their perinatal audit, the region already has a soft copy (Deputy Director #3).”

Feedback loops through audits, informal telephone communications, and more formalized supportive supervision and training were seen as available support systems to facilities.

“We have our planned quarterly supervision visits and supportive supervision visits…We do our best to visit some of the facilities. We are fortunate our current public health nurse is also a midwife, so she has that skill to coach and mentor newly posted midwives to do the right thing (District Health Manager#9).”

#### Funding constraints

Limited funding was highlighted as the major bottleneck to improving stillbirth recording and reporting. When funding is available, it is often from donors and earmarked.

“Funding is a big challenge. All our funding is from programmes so if a donor doesn’t have interest and all the money coming in is for vaccination, nutrition …, you will come up with priorities for the year and you will have perinatal and maternal death at the top, but we may go through the year and we would have done little to achieve the stated objectives because the funding was not there (Deputy Director #15).”

Funding limitations, respondents noted affects training, supportive supervision, coaching, and essential equipment. Most importantly, limited funds affect the frequency of stillbirth audits.

“…even moving from one facility to another for the stillbirth audit, the district will have to get fuel. Looking at the current situation, the district does not have any funds for those services. So sometimes you have to go on your own (Public Health Nurse #12).”

### DHIMS-2

#### Information sharing with other agencies.

Information captured in DHIMS-2 is only available to health information managers and senior officials within the public health sector. Other agencies such as statistical services or civil and vital registration, who play important roles in stillbirth monitoring do not have access to the DHIMS-2.

“Other agencies don’t have automatic access to the data. You need to be assigned an account before you can access the DHIMS-2. Often, it is Ghana Health Service who assigns, and it is not for everybody in the Service. It is specifically for data officers, health information officers and maybe managers of the health system who have access to it (Deputy Director #2).”

“…… every data from GHS is in the DHIMS-2, it is sensitive information. If the national statistical service will need it, they will have to put it officially in writing (Deputy Director #4).”

#### Community-level data

Currently the DHIMS-2 only captures information at the public health facility level. Some respondents flagged the importance of moving to a system that captures information from the community-level. Three respondents noted this is important for planning and delivering interventions within the peripheral of the district health system.

“The vital registration and statistics are not directly under the district health, so this is difficult to understand what is happening at the community level to plan interventions which we help deliver. DHIMS-2 could help with this (District Health Manager #10).”

#### Private sector engagement

The theme of private sector engagement was expressed by several of the RHD/DHD respondents. In Ghana, private facilities are autonomous. Though, they are mandated to report health data, including mortalities to the DHD, this is not always the case.

**“**In the event of a stillbirth, most private facilities do not report to the DHD meaning, a lot of stillbirth cases and other cases are missed (Deputy Director #2).”

One respondent flagged that in terms of data sharing on stillbirth, there were some challenges getting private sector facilities to submit monthly reports to the district-level. These challenges include staff attrition, limited skilled workforce and reporting does not bring profits.

“Since submitting reports does not generate revenue for the (private) facilities, sometimes you go to a facility, and they don’t even have a record officer who will submit reports. That is always a challenge (Health Information Officer #3).”

## Discussion

In understanding the experiences, perceptions, and attitudes of the RHD/DHD on stillbirth recording and reporting, we found that respondents understood the importance of stillbirth prevention and quality care, though there was varied understanding on what a stillbirth entails. Stillbirths were classified as fresh or macerated with limited references made to antepartum or intrapartum stillbirths. Data quality and DHIMS-2 were recurring themes for stillbirth data use. Support systems, funding constraints, sharing of data with other agencies and community-level data inclusion in DHIMS-2 were identified as enablers and barriers. Private sector engagement was a noted priority for respondents.

### Experiences, perceptions and attitudes

Leaders at the RHD/DHD were keenly aware of the importance of quality of care interventions such as ANC attendance and a skilled workforce in preventing stillbirths [20]. This finding was in line with other health workforce studies reported in Ghana and other similar settings [21–24]. An understanding of the importance of stillbirth prevention is crucial for district health mangers. District health managers who understand the significance of stillbirth prevention are more likely to prioritize data collection, analysis, and utilization, resulting in better-informed strategies and policies.

**Fig. 5:**
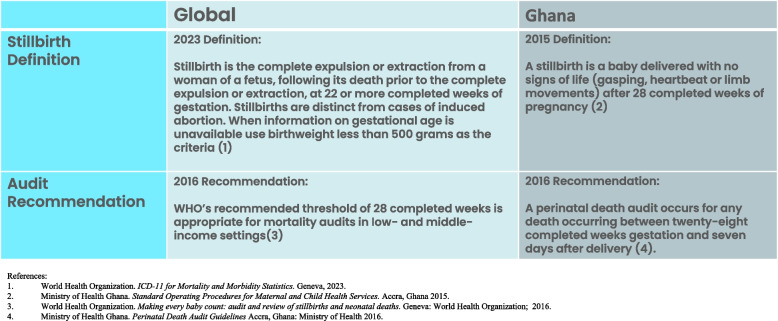
Stillbirth definition and audit recommendation in Ghana and globally

With the release of the WHO ICD-11, stillbirth is now defined as a baby born with no signs of life at 22 or more completed weeks of gestation [4]. The Ghana Health Service defines stillbirth as a baby delivered with no signs of life (gasping, heart beat or limb movements) after 28 completed weeks of pregnancy (Fig. 5) [25]. Interviews with the RHD and the DHD highlighted diverse understanding, within and between the different RHD/DHD cadres, on the definition of stillbirth. The lack of a universally understood definition of stillbirth affects how stillbirths are accurately recorded into routine health information management systems at the district-level and reported in national-level documents [26]. Further, non-standard application of the stillbirth definition has resulted in challenges in assessing stillbirth rates [2], thereby influencing prioritization, resourcing allocation and strategic planning based on gaps, and informing regional and district health management team support to health facilities.

Historically, classifying stillbirths in many LMICs has relied on fetal appearance based on assessment by the attending health care worker [27–29]. We found similar perspectives among regional and district health management teams in this study. Study participants classified stillbirth as macerated or fresh stillbirth with little reference to the timing around labor. Macerated stillbirth shows changes in skin i.e., soft-tissue changes, while fresh stillbirth does not. A study conducted at a tertiary hospital in the Ashanti region of Ghana found that using skin appearance is not an accurate proxy for stillbirth classification due to its subjective nature [27, 30, 31]. Misclassifications are likely to occur when a standard criterion is not applied between health care workers. The United Nations Inter-agency Group for Child Mortality Estimation (UN-IGME) and the WHO, as part of the ICD-11 release, are encouraging countries to move away from traditional visual assessments for timing of stillbirth, and towards a more accurate classification using absence of fetal heart activity on auscultation or ultrasound on admission to labour ward [4, 32]. This is intended to standardize the stillbirth definition and avoid misclassification.

Globally, 42% of stillbirths are intrapartum, with up to 50–70% in LMICs [2, 9]. Most stillbirths are preventable with quality of care interventions, which was well recognized by the RHD/DHD. Interventions such as monitoring mothers throughout pregnancy can prompt timely delivery of at-risk pregnancies [14] and improved intrapartum monitoring linked to timely action can avoid adverse pregnancy outcomes, including stillbirth [33]. Improving the accuracy of recording of fetal death, and including information around timing, will allow regional and district health teams to plan and track appropriate quality of care interventions to avert preventable stillbirths.

### Stillbirth data

DHIMS-2, an electronic data management system in Ghana, was established to aggregate routinely collected data across all public health facilities in the country, facilitate analysis, forecast required services, and evaluate performance of health care workers [34–37]. Information gathered from the DHIMS-2 is also used to formulate policies, evaluate programmes and allocate resources [38]. We discovered that when the national-level periodically shuts down DHIMS-2, health workers tend to report stillbirths by adding data from the previous month to the new month, leading to an increase in reported cases. This finding aligned with the known challenges on over-reporting of certain indicators from health facilities into health information management systems [39]. Similarly, in the absence of a universal online platform accessible by all health facilities, manual entry of data into health information management systems can be time-consuming, has shown to increase errors and has potential to decrease data quality which influences data analysis [37]. All these factors can impact decision-making, leading to ill-informed resource allocation and planning inefficiency at the regional and district level [40].

Early gestational stillbirth is defined as stillbirths occurring between 22 and 27 weeks. Late gestational stillbirths are fetal deaths occurring after 28 weeks. Some RHD/DHD members described the national mandate to conduct audits only if the fetus is over 28 weeks [41]. In Ghana, this is done due to the capacity of the health system to investigate third-trimester stillbirths or late fetal deaths. This national audit recommendation is aligned to global guidance from WHO, using an audit threshold of 28 completed weeks as appropriate for mortality audits in LMIC settings (Fig. 5) [42]. When audit thresholds start at 28 weeks, early gestational stillbirths are excluded. Whilst it may not be feasible to audit all stillbirths, excluding early gestational stillbirths may result in these deaths being perceived as having less value, which may result in them being missed from being counted in the data system [14]. This can potentially lead to under-reporting of the true burden of stillbirths in the routine health information management systems [43].

Literature surrounding blame of midwives and other health care workers in stillbirths is widely documented [44–47]. There were similar findings in this study with some RHD/DHD members blaming omission or gaps reported in stillbirth data on midwives. The trauma and guilt associated with stillbirth can cause health care workers to forgo recording and reporting stillbirth.

### Leadership and support mechanisms

Efforts to accurately record and report stillbirth data are often hampered by limited resources [2]. We found that funding constraints affect the frequency of audits, a systematic process to prevent future stillbirths [45]. Insufficient resourcing has been extensively documented as a barrier to audit implementation [48]. This hinders the monitoring, review and learning processes grounding perinatal audits; limits improvements to be made post-audits and contributes to gaps reported in routine health information management systems on the circumstances surrounding a death.

A recent study found that 21 countries (out of 66) required data on stillbirth at health facility or at the community-level be provided to the national statistics office, civil registration system, or equivalent bodies [17]. In exploring this further, we found that information captured within the DHIMS-2 is not easily accessible to other national agencies. Agencies requiring information from the DHIMS-2 need to undergo a formal process/request to GHS for the information. Understanding the magnitude of the stillbirth burden at country level, requires collaboration and triangulation of information across various data sources including the DHIMS-2, civil registration and vital statistics systems (CRVS) and the birth or death registries. With CRVS, the health sector can be a powerful ally in providing insights into births as well as the circumstances surrounding a death [49]. Ensuring timely access to information on stillbirth in DHIMS-2 can facilitate greater prioritization of the stillbirth agenda across agencies, foster inter-agency collaboration and drive investments into stillbirth reduction.

Over 70% of stillbirths in LMICs occur in community settings [50, 51]. Triangulating information from the community-level on stillbirths with information from health facilities provides a holistic picture of the true population burden of stillbirths. Interviewed RHD/DHD members flagged the importance of an integrated health information management systems which includes data on stillbirths from the community and the health facility level. Taking forward an integrated system was reported to optimize data timeliness and completeness though challenges were also reported on network connectivity and support systems for community health workers to report the data [52].

Private health facilities are increasingly becoming the first point of contact for the health system for many LMIC families including for maternal and child health service delivery, accounting for around 40% of antenatal and childbirth care contacts [53, 54]. In Ghana, private health facilities make up 40.2% of all health facilities, while government facilities (53.8%) and faith-based facilities (6%) complete the spectrum of service delivery actors [55]. In this study, although private health sector facilities within the Ashanti Region are mandated to report stillbirth data to the DHD, this did not always occur. Even when policies are in place, there are gaps in reporting from the private sector – a situation likely to be worse in the majority of countries without even a policy. This can potentially lead to under-reporting of stillbirths in DHIMS-2 and under-estimating the real burden of stillbirths since information from the private sector is not captured. Improving stillbirth data requires equal attention to public as well as private health facilities [2].

### Implications for practice

The findings from this study provide important information to inform improvements in stillbirth recording and reporting in the Ashanti Region of Ghana.

Immediate priorities for action include: first, the Ashanti RHD should organize a workshop for all DHD members within the region to sensitize the district health management teams on stillbirths and the types of stillbirths aligned to the national definition for stillbirths. This will facilitate consistent application of the stillbirth definition for recording and reporting. Second, revisit the national definition for stillbirths in Ghana, in light of the recent classification by WHO using the 22-week threshold. This ensures that all stillbirths are counted. Finally, review emerging global guidance on audit implementation to inform policy reforms.

Three long-term recommendations are proposed. These include the need to move towards a holistic digitalized DHIMS-2 for all health facilities. Two, integrate community-level data into DHIMS-2 to understand and manage district and regional-level support on stillbirths. Finally, whilst it is mandated that private sector facilities report data on stillbirth, measures should be put in place to ensure reporting by private facilities. This allows us to understand the scale, reach and true burden of stillbirths in the region.

### Limitations

Though Zoom is a highly suitable platform for collecting qualitative interview data, [56] we experienced some internet connectivity issues with some participants. The study was conducted in one of the four regions of Ghana not experiencing a decline in SBR. This might limit the generalizability of the findings. However, RHD/DHDs are regularly on rotation to different regions within the health system of Ghana. Additionally, findings from this study have been shared with the Ghana Health Service to ensure that recommendations in this study are scaled up to the rest of the country. Responses of the RHD/DHD may have been influenced by the presence of the first author. To address this, questions were asked repeatedly in a neutral manner and confidentiality was respected.

The first author has policy expertise in district health management, alongside an understanding of the issues on stillbirth recording and reporting. This may have influenced the thematic analyses.

## Conclusion

This study explored the critical role that DHMTs play in stillbirth recording and reporting. By understanding the importance of stillbirth recording and reporting, DHMTs can pave the way for evidence-informed decision-making, implement effective interventions, and deliver actions needed to achieve the global goal of 12 or fewer stillbirths per 1000 total births by 2030.

### Supplementary Information

**Additional file 1.** 

**Additional file 2.**

**Additional file 3.**

**Additional file 4.**

## Data Availability

The datasets used and/or analyzed during the study are available from the corresponding author on reasonable request.

## Abbreviations

ANC: Antenatal care
CRVS: Civil registration and vital statistics
COREQ: Consolidated criteria for reporting qualitative studies
DHD: District health directorate
DHIMS-2: District health information management systems 2
DHMT: District health management team
GHS: Ghana health service
ICD: International classification of diseases
LMICs: Low-and middle-income countries
LSHTM: London School of Hygiene and Tropical Medicine
MMR: Maternal mortality rate
NMR: Neonatal mortality rate
ODK: Open data kit
RHD: Regional health directorate
SBR: Stillbirth rate
UNICEF: United Nations Children’s Fund
UN-IGME: United Nations inter-agency group for child mortality estimation
WHO: World Health Organization
