# Supplementary material for: District health management and stillbirth recording and reporting: a qualitative study in the Ashanti Region of Ghana
Source: BMC Pregnancy Childbirth. 2024 Jan 29;24:91. doi: 10.1186/s12884-024-06272-x (PMC10826143; doi:10.1186/s12884-024-06272-x)
Supplement: Supplementary file 1 — Additional file 1. [file 12884_2024_6272_MOESM1_ESM.docx]

**Study tools: semi-structured questionnaire guide**

1. **Regional Health Directorate**

**Theme 1: Background**

1. Tell me about your current role in the Regional Health Directorate?

Probe:

- What does your job entail in relation to stillbirth or perinatal deaths?

**Theme 2: Experience, perception, attitudes**

1. Tell me about what you understand about what a stillbirth is?
2. What is needed to prevent a stillbirth?

Probe

- - Clinical knowledge and tools/equipment.
  - Why is it important to prevent stillbirths?

**Theme 3: Stillbirth Data Collection/Recording and Use of Stillbirth Data**

1. Can you tell me how does your region define a stillbirth?

Probe:

- 1. Do you know if the definition varies by region in Ghana? Does it vary by district in the Ashanti Region?

1. How is data on stillbirths collected and reported?

Probe:

- 1. What available tools or reviews do you have to support tracking stillbirth rates in the region?

Probe

- - Tell me about the templates or protocols or guidelines for reporting stillbirth in the region?
  - What about perinatal audits or integrated mechanisms with maternal mortality reviews or neonatal reviews? How often do these occur?
  1. Can you tell me about how the regional level is organized for data analysis, quality checks, interpretation, and use for stillbirths?

Probe:

- What is the process flow for stillbirth data? Who is involved? What is the communication and coordination between these individuals?
- When stillbirth data is reported at the facility and district-level, how is the data transferred to the regional-level?
- How is the data shared with the national level (Ghana Health Service or Ministry of Health)? What about other national agencies (e.g., national statistical office, CRVS stakeholders etc.?)
- Do you give feedback to districts for improvement? Are there learning sessions held with the districts to understand the data and address gap areas? Is this individually with regions or jointly?
- How are decisions made for funding allocation and programme allocation?

1. What functioning surveillance systems are there for monitoring the number of stillbirths in the region?

Probe

- Perinatal audit (MPDSR), Health Information Management Systems, District Health Information Software, civil registration and vital statistics, population surveys, others?
- Does the data reported to these surveillance systems include an indicator on stillbirth?

1. Looking at this visual prompt of stillbirth measurement over a period, tell me what you see?

**Theme 3: Leadership and support mechanisms**

1. Does recording and reporting a stillbirth matter?
2. Are there formal mechanisms in place in the region for the district health directorate in reducing stillbirths?

Probe

- Do these mechanisms include recording and reporting stillbirth?
- What about mentoring, supportive supervision, coaching and capacity building for stillbirths or perinatal health/child health or maternal health? How often do these occur?
- Do you know if women and community members are engaged in stillbirth education and awareness?

1. Can you tell me about the regional strategy/plan for Health? Does this include stillbirth reduction? What about improving stillbirth recording and reporting?
2. Is there funding allocated to improving maternal/ newborn health (and hence perinatal outcomes)?

Probe

- - Stillbirth reduction at the regional level?
  - What about stillbirth recording and reporting?
  - Are you aware of any other available funding to improve stillbirth data and reporting infrastructure at the district-level?

1. Are there any improvement projects or initiatives at the regional level to reduce stillbirths, or include a focus on stillbirth?

Probe

- - What about stillbirth recording and reporting?

**2: District Health Directorate**

*The interview guide for the DHD is adapted from the RHD interview guide with the addition of one question, marked as new.*

**Theme 1: Background**

1. Tell me about your current role in the District Health Directorate?

Probe

- What does your job entail in relation to stillbirth or perinatal deaths?

1. New. Can you tell me about how the district team is organized?

Probe

- How many DHD officers are assigned to the perinatal or child health? What about stillbirth? Does this include a focus stillbirth recording and reporting?

**Theme 2: Experience, perception, attitudes**

1. Tell me about what you understand about what a stillbirth is?
2. What is needed to prevent a stillbirth?

Probe

- - Clinical knowledge and tools/equipment.
  - Why is it important to prevent stillbirths?

1. What is your perception of the leaderships’ commitment towards prioritizing reducing stillbirths?

Probe

- District level and regional health directorate

**Theme 3: Stillbirth Data Collection/Recording and Use of Stillbirth Data**

1. Can you tell me how does your district define a stillbirth?

Probe:

- - Does it vary by district in the Ashanti Region?

1. How is data on stillbirths collected and reported?

Probe:

- - What available tools or reviews do you have to support tracking stillbirth rates in health facilities in the district?

Probe:

- - Tell me about the templates or protocols or guidelines for reporting stillbirth in the district?
  - What about perinatal audits or integrated mechanisms with maternal mortality reviews or neonatal reviews? How often do these occur?
  - Can you tell me about how the district level is organized for data analysis, quality checks, interpretation, and use for stillbirths?

Probe:

- What is the process flow for stillbirth data? Who is involved? What is the communication and coordination between these individuals?
- When stillbirth data is reported at the facility level, how is the data transferred to the district-level?
- How is the data shared with the regional health directorate?
- Do you give feedback to facilities for improvement? Are there learning sessions held with the districts to understand the data and address gap areas? Is this individually with facilities or jointly across facilities?
- How are decisions made for funding allocation and programme allocation?

1. What functioning surveillance systems are there for monitoring the number of stillbirths in the region?

Probe:

- Perinatal audit (MPDSR), Health Information Management Systems, District Health Information Software, civil registration and vital statistics, population surveys, others?
- Does the data reported to these surveillance systems include an indicator on stillbirth?

1. Looking at this visual prompt of stillbirth measurement over a period, tell me what you see?

**Theme 3: Leadership and support mechanisms**

1. Does recording and reporting a stillbirth matter?
2. Are there formal mechanisms in place in the district for facility health workers in reducing stillbirths?

Probe:

- What about mentoring, supportive supervision, coaching and capacity building for stillbirths or perinatal health/child health or maternal health? How often do these occur?
- Do these mechanisms include recording and reporting stillbirth?
- Do you know if women and community members are engaged in stillbirth education and awareness?

1. Can you tell me about the regional or district strategy/plan for Health? Does this include stillbirth reduction? What about improving stillbirth recording and reporting?
2. Is there funding allocated to improving maternal/ newborn health (and hence perinatal outcomes)?

Probe:

- - Stillbirth reduction at the regional or district level?
  - What about stillbirth recording and reporting?
  - Are you aware of any other available funding to improve stillbirth data and reporting infrastructure at the district-level?

1. Are there any improvement projects or initiatives at the district level to reduce stillbirths, or include a focus on stillbirth?

Probe

- - What about stillbirth recording and reporting?
