# Supplementary material for: District health management and stillbirth recording and reporting: a qualitative study in the Ashanti Region of Ghana
Source: BMC Pregnancy Childbirth. 2024 Jan 29;24:91. doi: 10.1186/s12884-024-06272-x (PMC10826143; doi:10.1186/s12884-024-06272-x)
Supplement: Supplementary file 3 — Additional file 3. [file 12884_2024_6272_MOESM3_ESM.docx]

## **Consent Form**

Title of Project: **Stillbirth recording and reporting: a qualitative study in the Ashanti Region of Ghana**

Name of researcher responsible for project: **Nana Afriyie Mensah Abrampah**

| **Statement** | **Please initial or thumbprint each box** |
| --- | --- |
| I confirm that I have read and understood the information sheet dated…………..for the above named study. I have had the opportunity to consider the information, ask questions and have these answered satisfactorily. |  |
| I understand that my consent is voluntary and that I am free to withdraw this consent at any time without giving any reason and without my/the participant’s legal rights being affected. |  |
| I understand that relevant sections of my data collected during the study may be looked at by authorised individuals from LSHTM, where it is relevant to my/the participant’s taking part in this research. I give permission for these individuals to have access to these records. |  |
| I understand that data about/from me/the participant may be shared via a public data repository or by sharing directly with other researchers, and that I will not be identifiable from this information |  |
| I agree to me/the participant taking part in the above-named study. |  |
| I have read the foregoing information through the project information sheet, or it has been read to me in a language that I understand. I have had the opportunity to ask questions; and any question I have asked has been answered to my satisfaction. I consent voluntarily to participate in this study and understand that I have the right to withdraw from the study at any time without in any way, it affecting my further medical provision. |  |
| I acknowledge that I have read or have had the purpose and contents of the Participants’ Information Sheet read and all questions satisfactorily explained to me in a language I understand English/Asante Twi. I fully understand the contents and any potential implications as well as my right to change my mind (i.e. withdraw from the research) even after I have signed this form.  I voluntarily agree to be part of this research. |  |

|  |  |  |
| --- | --- | --- |

Printed name of participant Signature of participant Date

(or thumbprint/mark if unable to sign)

STATEMENT OF WITNESS

The participant/representative is unable to sign. As a witness, I confirm that all the information was given and the participant/representative consented to taking part.

I was present when the purpose and contents of the Participant Information Sheet was read and explained satisfactorily to the participant in the language, he/she understood, English/ Asante Twi.

I confirm that he/she was given the opportunity to ask questions/seek clarifications and same were duly answered to his/her satisfaction before voluntarily agreeing to be part of the research.

|  |  |  |
| --- | --- | --- |

Printed name of witness Signature Date

INVESTIGATOR STATEMENT AND SIGNATURE

I certify that the participant has been given ample time to read and learn about the study. All questions and clarifications raised by the participant have been addressed.

|  |  |  |
| --- | --- | --- |

Printed name of person obtaining consent/researcher Signature Date
