# Supplementary material for: District health management and stillbirth recording and reporting: a qualitative study in the Ashanti Region of Ghana
Source: BMC Pregnancy Childbirth. 2024 Jan 29;24:91. doi: 10.1186/s12884-024-06272-x (PMC10826143; doi:10.1186/s12884-024-06272-x)
Supplement: Supplementary file 4 — Additional file 4. [file 12884_2024_6272_MOESM4_ESM.docx]

Project information leaflet for regional and district health officers

The Information Sheet provides information about the research for participants to make an informed decision of whether to participate in the study or not. It outlines the nature of the research, what the research involves, risks, benefits, compensation.

**Title of Study:** Stillbirth recording and reporting: a qualitative study in the Ashanti Region of Ghana

**Introduction:** My name is Nana Afriyie Mensah Abrampah. I am a PhD student at the London School of Hygiene and Tropical Medicine, and I am conducting the above study as principal investigator. My email address is [nana.mensah-abrampah@lshtm.ac.uk](mailto:nana.mensah-abrampah@lshtm.ac.uk).

**Background and Purpose of research**: Many women experience a stillbirth. Every 16 seconds, one stillbirth occurs. Three in four stillbirths occur in sub-Saharan Africa or Southern Asia. Though the burden is high, stillbirths are preventable with the right quality care provided by the health workers. Stillbirths can be fresh or macerated, with gestational age, birthweight and length providing an indication of whether a stillbirth has occurred. Stillbirths are often not counted or misclassified due to similarities with other fetal or neonatal deaths.

Better stillbirth measurement and reporting may help to provide reliable data on stillbirth. This in turn can increase investments and identify appropriate interventions to reduce stillbirths. The regional health directorate and district health officers play a role in improving stillbirth reporting as they attend to data emerging from the facility-level and other data sources at the district-level (civil registration and vital statistics, and population surveys). The district-level also serves as the link between the national and facility-levels for monitoring of priority health conditions. We want to understand stillbirth recording and reporting amongst the regional health directorate team and the district health management team. We are therefore doing a study to understand this.

**Nature of research:** You have been chosen to participate in this study because you are a regional health directorate representative in the Ashanti Region or district health officer working in [insert district name] responsible for management of health, monitoring and evaluation, surveillance or Reproductive, Maternal, Child and Adolescent Health (RMNCAH) within this district. District health officers with these responsibilities are being invited to participate. The total number of district health officers recruited for this study will be around 16 participants and all members of the regional health directorate leadership team.

The interview will include questions on experience, perception, and attitude of the regional health directorate (who oversee the district health management team), and the district health management teams on stillbirth recording and reporting. In addition, given that a key role of district health management teams is routine data collection, we will explore data use on stillbirth at the district level and available support mechanisms to facilitate stillbirth recording and reporting.

**Duration /what is involved:** The interview is optional. It is up to you to decide whether or not to take part. If you do, you will be given this information sheet to keep and be asked to sign a consent form. For you to take part, you must agree to participate in the study. You are still free to withdraw at any time and without giving a reason. A decision to withdraw at any time, or a decision not to take part, will not affect your standing in the region/district.

If you choose to take part, the interview will take 45 minutes to 1 hour. The interview will be done by the researcher online. If you agree to take part in the study, we will expect you to be available for the interview and to answer the questions posed by the researcher truthfully.

If you choose to take part, the interview will take 45 minutes to 1 hour. The interview will be done by a researcher over Zoom. Please see the attached Zoom privacy document for further information.

**Potential Risks:** If you choose to participate in the study, you will need to be available for one session for 45 minutes to 1 hour. Some people may find it difficult to find the time for this session. Other health workers may approach you and have queries about the study. Should you be approached, please do share this information leaflet or refer the health worker to the researcher.

**Benefits:** We cannot promise the study will help you but the information we get might help improve the measurement and reporting of stillbirths.

**Costs and compensation:** There will be no direct costs incurred or provided by participating in the study.

**Confidentiality:** If you join the study, some parts of data collected for the study will be looked at by authorised persons from the London School of Hygiene & Tropical Medicine. All will have a duty of confidentiality to you as a research participant and nothing that could reveal your identity will be disclosed outside the research site.

All information which is collected about you during the course of the research will be kept strictly confidential. Any information about you which leaves the health centre will have your name and address removed so that you cannot be recognized from it.

Information that identifies you will not be collected. Only demographic information, including health worker cadres, facility-type, and years in service will be collected. All information provided during the interview will be secured safely on a computer. All information collected from you will be kept confidential.

**Voluntary participation/withdrawal:** Participation is voluntary, and participants have the right to decline to participate and also withdraw from the study at any time without penalty and without having to give any reasons.

**Outcome and Feedback:** The researcher intends to write a report of the results and publish them in a scientific journal. The researcher will hold a virtual meeting over zoom to present the results of the study, which you will be invited to attend. You will not be identified in any report, publication or presentation.

**Funding information**: There is no funding agency for the study. The London School of Hygiene and Tropical Medicine, UK, is organizing this study.

**Sharing of participants Information/Data:**  The principal investigator will have access to all the data.

**Provision of Information and Consent for participants:** A copy of the information sheet and consent form will be made available to you to sign if you agree to participate in the study.

**Who to Contact for Further Clarification/Questions:** If you have a concern about any aspect of this study, you should ask to speak to the researcher who will do their best to answer your questions, please email [nana.mensah-abrampah@lshtm.ac.uk](mailto:nana.mensah-abrampah@lshtm.ac.uk) . If you remain unhappy and wish to complain formally, you can do this through Debra.Jackson@lshtm.ac.uk or Nana Abena Apatu (ethics.research@ghsmail.org).
